# Catalytic response and molecular simulation studies in the development of synthetic routes in trimeric triaryl pyridinium type ionic liquids

**DOI:** 10.1038/s41598-023-31476-0

**Published:** 2023-03-17

**Authors:** Ramalingam Tamilarasan, Annadurai Subramani, G. Sasikumar, Pandurangan Ganapathi, S. Karthikeyan, Sasikumar Ponnusamy, Salim Albukhaty, Mustafa K. A. Mohammed, Zaidon T. Al-aqbi, Faris A. J. Al-Doghachi, Duha S. Ahmed, Yun Hin Taufiq-Yap

**Affiliations:** 1 Department of Chemistry, Vel Tech Multi Tech Dr. Rangarajan Dr. Sakunthala Engineering College, Avadi, Chennai, India; 2 Department of Biochemistry, Dwaraka Doss Goverdhan Doss Vaishnav College, Chennai, Tamilnadu 600106 India; 3 Department of Chemistry, Mohamed Sathak College of Arts & Science, Sholinganallur, Chennai, India; 4 Department of Physics, Periyar University Centre for Post Graduate and Research Studies, Dharmapuri, 636 701 India; 5 Department of Physics, Saveetha School of Engineering, (SIMATS), Thandalam, Chennai, 602 105 India; 6 College of Medicine, University of Warith Al-Anbiyaa, Karbala, Iraq; 7 Radiological Techniques Department, Al-Mustaqbal University College, 51001 Hillah, Babylon, Iraq; 8 College of Agriculture, University of Misan, Al-Amara, Misan 62001 Iraq; 9 Department of Chemistry, Faculty of Science, University of Basrah, Basrah, 61004 Iraq; 10 Applied Science Department, University of Technology, Baghdad, 10011 Iraq; 11 Catalysis Science and Technology Research Centre, Faculty of Science, Universiti Putra Malaysia, 43400 Serdang, Selangor Malaysia; 12 Faculty of Science and Natural Resources, University Malaysia Sabah, 88400 Kota Kinabalu, Sabah, Malaysia

**Keywords:** Biochemistry, Biological techniques, Chemistry

## Abstract

Under conventional and silica-supported Muffle furnace methods, water-soluble substituted trimeric triaryl pyridinium cations with various inorganic counter anions are synthesized. The solvent-free synthesis method is superior to the conventional method in terms of non-toxicity, quicker reaction times, ease of workup, and higher yields. Trimeric substituted pyridinium salts acted as excellent catalytic responses for the preparation of Gem-bisamide derivatives compared with available literature. To evaluate the molecular docking, benzyl/4-nitrobenzyl substituted triaryl pyridinium salt compounds with VEGFR-2 kinase were used with H-bonds, π–π stacking, salt bridges, and hydrophobic contacts. The results showed that the VEGFR-2 kinase protein had the most potent inhibitory activity. Intriguingly, the compound [NBTAPy]PF_6_^-^ had a strongly binds to VEGFR-2 kinase and controlled its activity in cancer treatment and prevention.

## Introduction

Ionic liquids (ILs) have recently attracted the attention of chemists because of their unique properties, such as non-volatility, non-flammability, chemical stability, a high electrochemical window, and a large liquid-state heating rate^1–6^. Cations, which are commonly organic, and anions, which are generally inorganic, are present in ILs to prevent the salts that result from packing completely. As a result, IL resists crystallization and maintains its liquid state across a broad temperature range^7–11^. Aqueous and organic solvents are often used in solvolysis. Additionally, it is feasible to polarize or saturate non-polar substances using ILs. In light of this, ILs are receiving more attention as a potential substitute for the conventional volatile, unreliable molecular solvents used in organic processes and catalysis. Dipolar compounds can to be modified and altered, which makes them helpful in the disciplines of conductive ion species, catalytic activity, and organic process modification. One of the best things about ionic liquids that have been changed and given new functions is that they can be replaced^12^. Environmental awareness has heightened the need for scientists to develop "reaction systems" that are cleaner and more efficient^13–16^. One of the best ways to avoid using volatile organic solvents is to use room-temperature ionic liquids as the reaction medium^17,18^. Ionic liquids are becoming more popular as green synthesis solvents that can be used instead of traditional organic solvents in many different chemical processes^19–22^. Room temperature ILs carry out a variety of unique tasks because of their special properties, which include vital conducting behaviors, high chemical and thermal stability, non-flammability, and shallow vapour pressure. This broadens the applicability of ionic liquids^23^. Imidazolium-based ILs are one of the types of ILs whose chemistry has received the most attention^24,25^. This is because of how easy it is to change the chemical and physical properties of imidazolium-based IL by changing the nitrogen substitution in the ring and using different anions^26,27^. Research on azolium-based ILs has shown that triazolium-based ILs, particularly 1, 2, 4-triazolium based ILs, are less studied in terms of synthesis and use^28–30^.

Zhao et al*.* say that immobilizing homogeneous catalyst has become essential for making it easier to get the catalyst out and then use it again^31^. Hyperbranched polymers with triazine linkages based on polyethylene oxide serve as polymer electrolytes^32^. Styrene was selectively oxidized with a transition metal and imidazolium halide, producing a product with a lower yield^33^. A quantitative yield was obtained by acetylating several substituted aryl aldehydes with acidic anhydride in the presence of catalytic amounts of ILs^24^. ILs also function more effectively as Lewis acids^34^.

With different counter anions, the imidazolium/pyridinium salt's characteristics can either improve or suppress catalytic properties^1,35^. Due to their therapeutic potentials, such as their anti-inflammatory properties, pyrazole and its derivatives have become significant participants among other heterocyclic compounds^36–38^. Some studies demonstrated that pyrazolium/pyridinium, imidazolium, and other materials, such as nitrogen found in phosphane and ligands, may be combined to increase the stability of the molecule, which contains reusable active moieties^39–42^. One-pot synthesis of 1, 3, 5-triazine and its derivatives from aryl amine and formaldehyde in the presence of tetramethyl guanidinium salt. Triaryl triazine compound, tested in vitro for antimicrobial activity against mycobacterium tuberculosis, it showed excellent response^43^.

The most stable protective aldehydes are gem-bisamides or bisurides, significant amide derivatives. Because of its unique properties, the amide bond's derivatives, such as biochemicals, polymer building blocks, and stable synthetic intermediates, can play a wide range of essential roles^44^. They are essential building blocks for adding a gem-diaminoalkyl residue to several molecules that have pharmacological or physiological effects^45–47^. Biologically powerful medicines have been synthesized using bisamides as ligands in Ullmann coupling processes^48^. As a general rule, two amides and one aldehyde are combined directly using catalysts such as CC-activated DMSO, boric acid, SiO_2_-MgCl_2_, ZnCl_2_, SiO-bonded S-sulfuric acid, nano-SnCl_4_.SiO_2_, molybdate-silica sulphuric acid, and polyphosphoric acid^49–51^. Dimeric, trimeric, and tetrameric pyridinium cations with bromide counter anion play a crucial role in detecting various anions in an aqueous environment reported^52^. Pravin and coworkers reported that the synthesis of triaryl pyridine under solvent-free conditions is catalyzed by using a heavy metal derivative such as bismuth triflate^53^. Recent studies mentioned that the 3,4,5-triaryl pyridine system showed particular medicinal interest due to its close structural coincidence with the acceptable photodynamic cell and thiopyrylium cancer therapeutic moieties^54^. Laine and coworkers reported that substituted triaryl derivatives act as novel materials in photochemistry for the conversion of solar energy into chemical reactions^55^.

Herein, we report a conventional/solvent-free silica-supported synthesis of a trimeric triaryl-substituted pyridinium cation with different counter anions. We have examined the catalytic properties of our trimeric triaryl substituted pyridinium salts for the one-pot synthesis of Gem-bisamide and its derivatives using conventional and muffle furnace approaches. We investigated the molecular simulation and binding affinities of trimeric triaryl substituted pyridinium compounds as drug candidate molecules against selected macromolecular receptors, such as H-bonding, π-π* stacking, salt bridges, and hydrophobic contacts.

## Results and discussion

**Figure 1:**
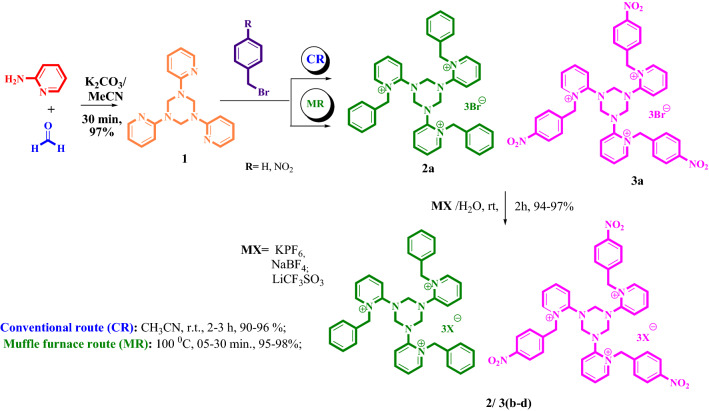
Synthetic route of substituted trimeric triaryl pyridinium salts.

The one-pot reaction (Fig. 1) between 2-amino pyridine (C_5_H_6_N_2_, 1.0 equi; 3.187 × 10^–2^ mmol) and formaldehyde (1.0 equi; 3.347 × 10^–2^ mmol) was carried out with both the dehydrated potassium carbonate (K_2_CO_3_, 1.0 equi; 3.014 × 10^–2^ mmol) and the addition of 30 mL of dry acetonitrile (CH_3_CN) as only a solvent, resulting in the creation of substituted pyridine derivative **1**^56^. To provide the purest form of substituted triazine derivatives of pyridine **1** in 98% yields, it is further purified using chloroform (CHCl_3_): hexane (C_6_H_14_) (20:80) chromatography. The *N-*alkylation was finished by of mixing 1.0 equi of trimeric triaryl pyridine derivative **1** (2.673 × 10^–3^ mmol) with benzylbromide (C_7_H_7_Br)/4-nitrobenzylbromide (3.05 equi; 1.304 × 10^–3^ mmol). The presence of 20 mL of dry CH_3_CN for 2–3 h in such a reflux condenser condition provides *N-*alkylated substituted pyridinium bromide derivative **2a/3a** in terms of yield.

Then, the *N-*alkylation reaction was carried out in a Muffle furnace at 100 $^\circ$C using a solvent-free silica-assisted reaction. Due to the solid silica-supported technique's quicker reaction time and higher yield, it is considered superior to the conventional approach. During the *N-*alkylation reaction, the conventional method is 15 times slower than the solvent-free silica-supported procedure; 4-nitrobenzyl bromide reacts substantially more quickly than benzyl bromide. Ionic liquids, in general, have varied physical as well as chemical merits depending on the counter anion. Therefore, studying both of the physical as well as the chemical an anion exchange process can be carried out in 20 mL of deionized water while it is stirred for 2 h. Using a variety of inorganic compounds that include counter ions, such as K_4_PF_6_, NaBF_4_, and LiCF_3_SO_3_, one can obtain the anion exchange products **2/3 (b-d)** in 94–97% of yields (Table 1).

**Table 1:** Gem-bisamide derivatives synthesized with the use of various concentrations of benzyl-bridged trimeric pyridinium salts **2 (a-c).**

S.No	Catalyst	Derivative	Concentration of benzyl bridged trimric pyridinium salts											
			2.405 × 10^–2^ mmol				4.811 × 10^–2^ mmol				7.216 × 10^–2^ mmol			
			CR		MR		CR		MR		CR		MR	
			Time	Yield %	Time	Yield %	Time	Yield %	Time	Yield %	Time	Yield %	Time	Yield %
**1**	[BTAPy] Br^–^, **2a**	**4**	110	76	55	78	85	81	45	84	60	86	35	91
		**4a**	60	80	30	83	35	85	20	88	15	90	10	95
		**4b**	80	79	40	81	55	84	30	86	30	89	20	94
		**4c**	100	77	50	79	75	82	40	84	50	88	30	92
		**4d**	135	74	65	76	110	79	55	83	85	87	45	91
**2**	[BTAPy] PF_6_^–^ **2b**	**4**	120	73	65	76	95	78	55	82	70	83	45	88
		**4a**	70	77	40	80	45	83	30	86	20	88	20	95
		**4b**	90	75	50	78	65	81	40	83	40	86	30	92
		**4c**	110	71	60	74	85	76	50	79	60	82	40	87
		**4d**	145	69	75	72	120	76	65	77	95	81	55	86
**3**	[BTAPy] BF_4_^–^ **2c**	**4**	130	71	80	74	105	78	65	79	80	84	55	89
		**4a**	80	74	50	77	55	79	40	82	30	86	30	91
		**4b**	100	72	60	75	75	77	50	80	50	82	40	87
		**4c**	120	70	70	73	95	75	60	78	70	80	50	85
		**4d**	155	67	85	70	130	72	75	75	105	78	65	87
**4**	[BTAPy]CF_3_SO_3_^–^ **2d**	**4**	135	68	85	71	110	73	70	77	85	78	60	89
		**4a**	85	70	55	73	60	75	45	78	35	80	35	85
		**4b**	105	67	65	70	80	72	55	74	55	79	45	88
		**4c**	125	63	75	66	100	68	65	73	75	78	55	84
		**4d**	165	61	90	64	135	70	80	69	110	81	70	87

### Catalytic activity

**Figure 2:**
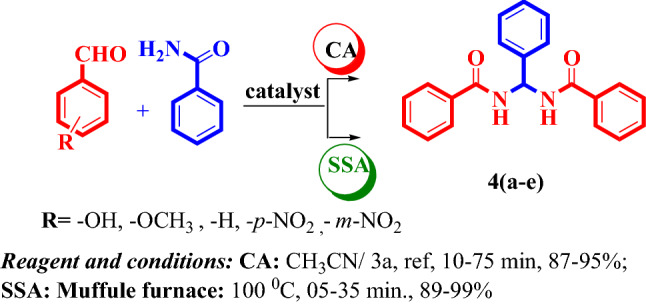
One-pot synthesis of substituted gem-bisamides derivatives under multiple approach.

Various catalytic methods have been used to prepare gem-bisamides, which has been thoroughly described in the literature. Abdolkarim Zare et al*.* reported on the preparation of gem-bisamides using nano-[DSPECDA][HSO_4_] as an expensive catalyst for two hours at 90 °C^57^. To obtain a low yield, gem-bisamides were made from different aryl aldehydes and benzamide in the presence of 20 mol% Agnps@modified TEOS xerogel, toluene, and a high temperature of 110 °C for 3–4 h^58^. Therefore, we synthesized the gem-bisamides with a non-toxic catalyst under typical conditions and with a shorter reaction time based on the literature (Fig. 2).

The preparation of gem-bisamides under ambient reaction conditions (2.069 × 10^–2^ mmol) with an optimal concentration of nitrobenzyl bridged trimeric pyridinium salt catalyst yielded good results, as shown in Table 2. Substituted benzyl/nitrobenzyl-bridged trimeric pyridinium bromide **2a/3a** is dispersed in water with a measured quantity of salts including NaBF_4_, K_4_PF_6_ and LiCF_3_SO_3_ at ambient conditions for 60 min with stirring to give products of anion exchange **2–3 (b–d)** in a 94–97% yield. We use spectroscopic equipment to examine each of our prepared compounds (see the supporting information file).

**Table 2:** Gem-bisamide derivatives synthesized with the use of various concentrations of 4-nitrobenzylbromide-bridged trimeric pyridinium salts **3(a–c).**

S.No	Catalyst	Derivative	Concentration of 4- nitrobenzyl bridged trimeric pyridinium salts											
			2.069 × 10^–2^ mmol				4.138 × 10^–2^ mmol				6.208 × 10^–2^ mmol			
			CR		MR		CR		MR		CR		MR	
			Time	Yield%	Time	Yield%	Time	Yield%	Time	Yield%	Time	Yield%	Time	Yield %
1	[NBTAPy]Br^–^ **3a**	4	80	81	40	87	60	90	20	93	30	93	10	95
		4a	30	88	20	84	20	89	10	90	10	91	05	94
		4b	50	79	25	86	30	90	13	92	15	94	07	95
		4c	70	81	35	90	50	93	17	95	25	94	10	97
		4d	105	79	55	89	85	92	27	96	43	93	27	98
2	[NBTAPy]PF_6_^–^, **3b**	4	90	85	45	85	70	89	25	91	40	92	15	96
		4a	40	75	25	82	20	88	15	91	10	90	10	97
		4b	60	80	30	83	40	91	18	90	25	93	12	93
		4c	80	73	40	89	60	91	22	93	35	93	15	96
		4d	105	81	60	86	95	91	32	94	53	92	32	97
3	[NBTAPy]BF_4_^–^ **3c**	4	100	73	50	82	70	86	30	90	50	91	20	94
		4a	50	75	30	80	30	93	20	89	15	91	15	96
		4b	60	73	35	84	50	89	23	88	35	90	17	92
		4c	80	80	45	88	70	89	27	92	45	91	20	93
		4d	115	71	65	82	105	90	37	94	63	91	37	95
4	[NBTAPy]CF_3_SO_3_^–^ **3d**	4	105	73	55	80	75	84	35	89	55	90	25	90
		4a	55	81	35	79	35	85	25	88	20	89	25	93
		4b	65	88	40	80	55	87	28	89	45	91	22	91
		4c	85	79	50	86	75	88	32	91	50	90	25	92
		4d	120	81	70	81	110	89	42	90	65	89	42	94

We proved the one-pot synthesis of gem-bisamides from various aldehydes under the existence of CH_3_CN using a low concentration of our substituted benzyl/nitrobenzyl bridged trimeric pyridinium salt 2–3 (a-d) under conventional/Muffle furnace conditions for 05–165 min to produce a yield of 80–98%, as shown in Tables 1 and 2. Nitrobenzyl bridged trimric pyridinium salts **2 (a–d)** exhibited superior catalytic activities than the benzyl bridged trimric pyridinium salts derivatives. We have examined the catalytic activity of various counter anions, including Br, BF_4_, PF_6_, and CF_3_SO_3_, which also include trimric pyridinium salts. Among these, bromide containing trimric pyridinium salts derivatives showed excellent catalytic behavior than the other. We determined that the optimum concentration of nitrobenzyl-bridged trimric pyridinium bromide is 6.208 × 10^–2^ mmol for preparing gem-bisamides. Under the silica-supported method, the 4-Nitrobenzylbromide bridged trimeric pyridinium salt takes 05 min to complete. Because of its quick reaction times, higher yield without using solvents, and environmental friendliness, the catalyst shows potential.

### Molecular docking with VEGFR2 (vascular endothelial growth factor receptor 2)

The activity of endothelial cells in vasculogenesis and angiogenesis is mostly regulated by vascular endothelial growth factor-A (VEGF-A, also called vascular permeability factor)^59–61^. Through VEGF receptors, vascular endothelial growth factor operates as a ligand^62^. The VEGF receptors VEGFR-1, VEGFR-2, and VEGFR-3 exist in humans in at least three different forms. In humans, VEGFR-2 is the predominant VEGFR. Both lymphatic and vascular endothelial cells exhibit high levels of this expression. Additionally, megakaryocytes, hematopoietic stem cells, brain cells, and other cancer cells express VEGFR-2.

### Validation on the active sites of VEGFR-2 kinase

**Figure 3:**
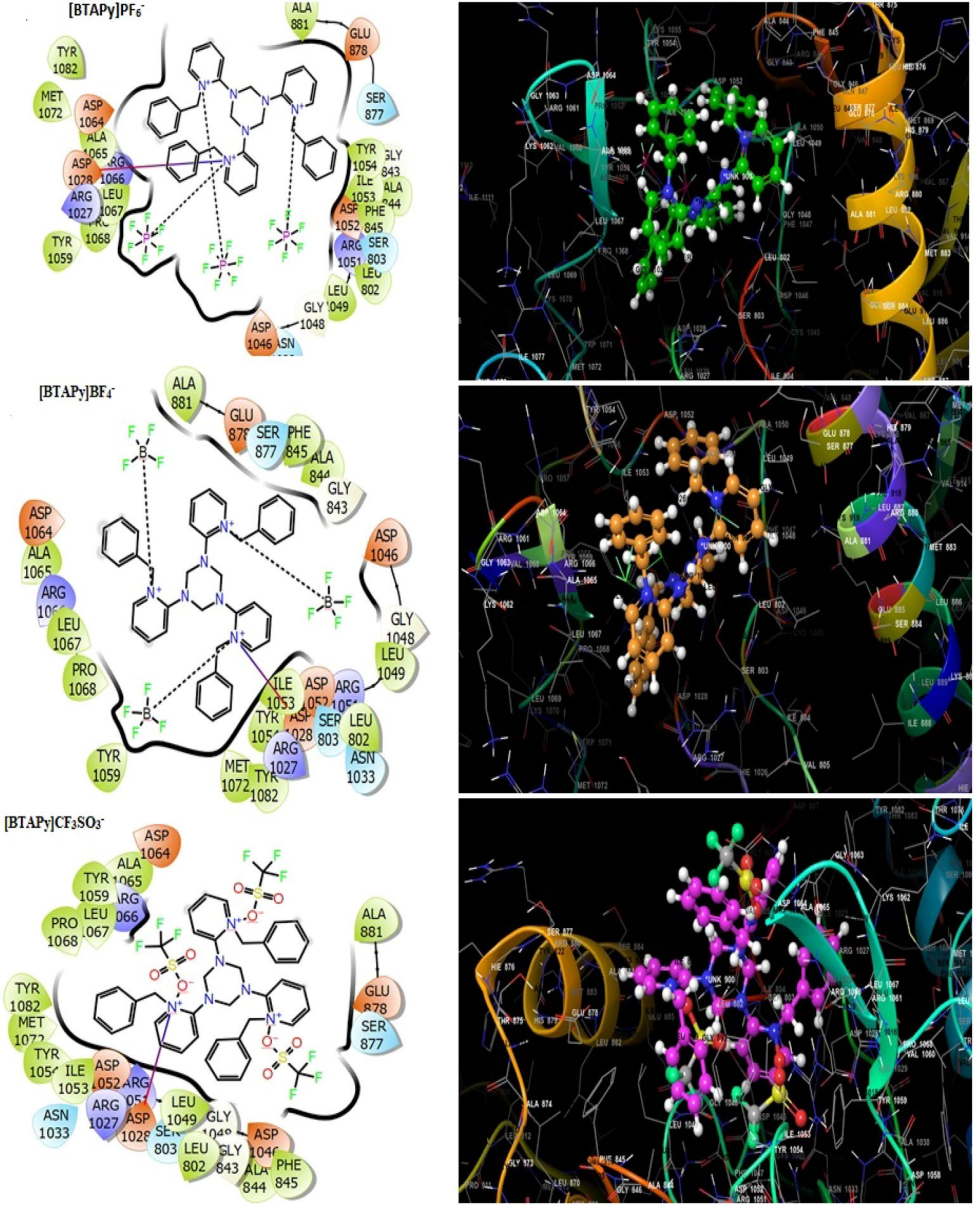
Molecular docking studies of synthesized compounds such as [BTAPy]PF_6_^–^, [BTAPy]BF_4_^–^ and [BTAPy]CF_3_SO_3_^–^ with VEGFR2 receptor.

**Figure 4:**
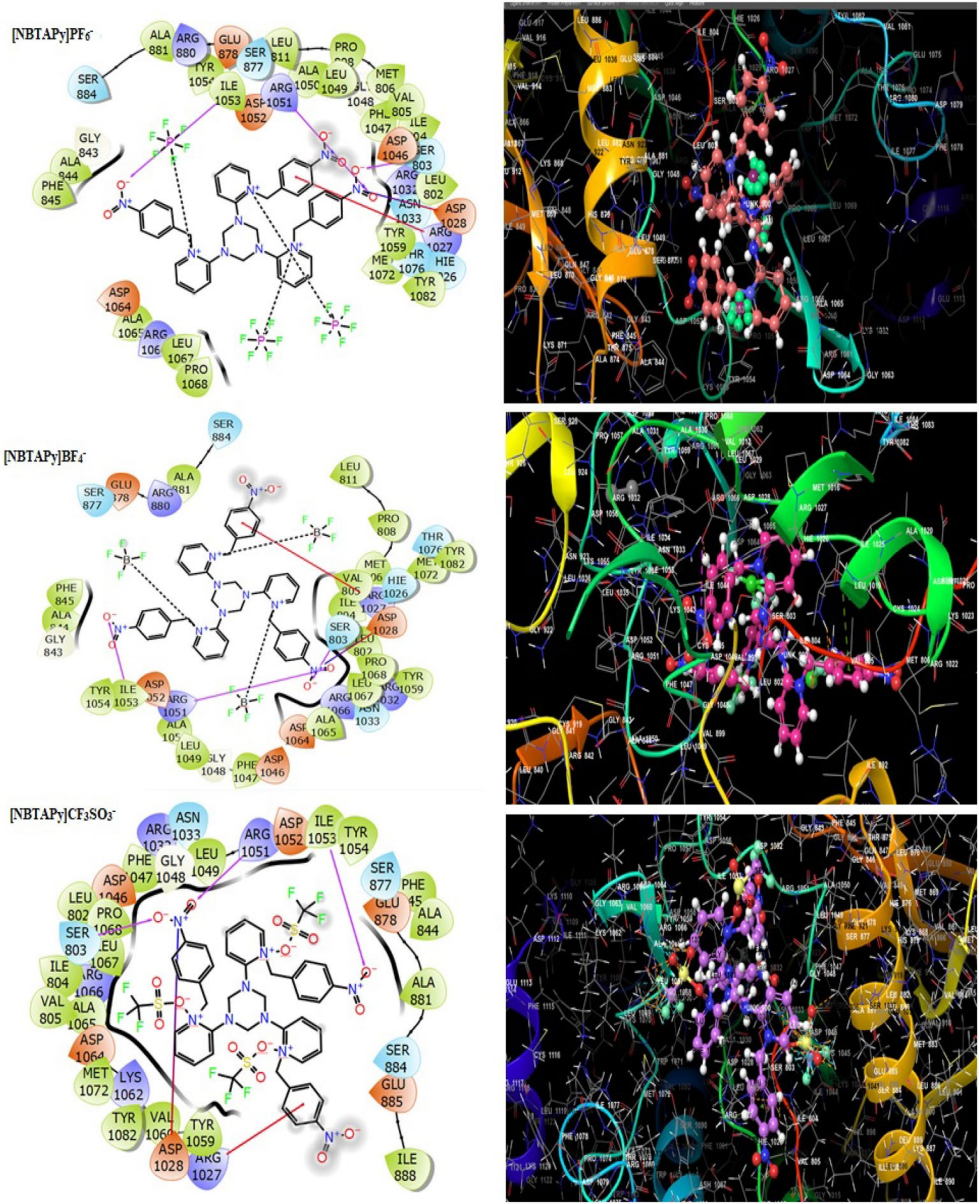
Molecular docking studies of synthesized compounds such as [NBTAPy]PF_6_^–^, [NBTAPy]BF_4_^–^and [NBTAPy]CF_3_SO_3_^–^ with VEGFR2 receptor.

The compound-protein interactions may be understood using molecular docking analyses, which can support our experimental findings. In Figs. 3 and 4, the ideal compound-target protein interaction site was shown, and data were collated in Table 3. Compounds Benzyl triarylpyridinium hexa flouro phaspate [BTAPy]PF_6_^–^, Benzyl triarylpyridinium tetra flouro boran [BTAPy]BF_4_^–^, Benzyl triarylpyridinium triflouro methane sulphonate [BTAPy]CF_3_SO_3_^–^, Nitrobenzyl triarylpyridinium hexa flouro phaspate [NBTAPy]PF_6_^–^, Nitrobenzyl triarylpyridinium tetra flouro boran [NBTAPy]BF_4_^–^ and Nitrobenzyl triarylpyridinium triflouro methane sulphonate [NBTAPy]CF_3_SO_3_^–^ observed docking score values of −6.327, −6.247, −6.546, −7.327, −7.154, and −7.099 kcal/mol, respectively. The binding docking scores were variable due to the structural variation of the triaryl pyridinium ionic liquid compounds. The docking score data showed that all compounds were correctly positioned in the hydrophobic location and strongly interacted with the VEGFR-2 kinase receptor through interactions with hydrogen bonds, hydrophobic molecules, and pi-pi stacking. The compound [NBTAPy]PF_6_^–^ displayed the highest docking score, which was influenced by a hydrogen bond with the residue ARG 1051, ILE 1053, SER 803, pi–pi interaction with ARG 1027, and numerous hydrophobic contacts, including LEU 802, MET 806, PRO 811, PHE 845, ALA 844, ALA 881, ILE 804, VAL 805, ALA 1050, ALA 1065, LEU 1067, PRO 1068, PHE 10 According to the aforementioned information, the compound [NBTAPy]PF_6_^–^ significantly binds and controls VEGFR-2 kinase activity in therapeutic approaches and cancer prevention.

**Table 3:** Molecular docking score of [BTAPy]PF_6_^–^, [BTAPy]BF_4_^–^, [BTAPy]CF_3_SO_3_^–^, [NBTAPy]PF_6_^–^, [NBTAPy]BF_4_^–^ and [NBTAPy]CF_3_SO_3_^–^ compound with VEGFR2 receptor.

Compounds	Docking score kcal.mol^−1^	Active sites with a mode of interaction			
		H-bond	π-π stacking	Salt bridge	Hydrophobic contacts (Cutoff at 5 Å)
**[BTAPy]PF**_**6**_^**–**^	−6.327	–	–	ASP 1028	GLY 843, PHE 845, ALA 881, LEU 802, ALA 844, LEU 1049, ILE 1053, TYR 1054, TYR 1059, ALA 1065, LEU 1067, PRO 1068, MET 1072, TYR 1082
**[BTAPy]BF**_**4**_^**–**^	−6.247	–	–	ILE 1053	PHE 845, ALA 881, LEU 802, ALA 844, LEU 1049, ILE 1053, TYR 1054, TYR 1059, ALA 1065, LEU 1067, PRO 1068, MET 1072, TYR 1082
**[BTAPy]CF**_**3**_**SO**_**3**_^**–**^	−6.546	–	–	ASP 1028	LEU 802, PHE 845, ALA 844, ALA 881, LEU 1049, ILE 1053, TYR 1054, TYR 1059, ALA 1065, LEU 1067, PRO 1068, MET 1072, TYR 1082
**[NBTAPy]PF**_**6**_^**–**^	−7.327	ARG 1051,ILE 1053, SER 803	ARG 1027	ASP 1028	LEU 802, MET 806, PRO 808, LEU 811, PHE 845, ALA 844, ALA 881, ILE 804, VAL 805, ALA 1050, ALA 1065, LEU 1067, PRO 1068, PHE 1047, LEU 1049, ILE 1053, TYR 1054, MET 1072, TYR 1082, VAL 1060, TYR 1059
**[NBTAPy]BF**_**4**_^**–**^	−7.154	ARG 1051,ILE 1053	VAL 805	ASP 1028	LEU 802, MET 806, PRO 808, LEU 811, PHE 845, ALA 844, ALA 881, ILE 804, VAL 805, ALA 1065, LEU 1067, PRO 1068, PHE 1047, LEU 1049, ILE 1053, TYR 1054, MET 1072, TYR 1082, VAL 1060, TYR 1059
**[NBTAPy]CF**_**3**_**SO**_**3**_^**–**^	−7.099	ARG 1051,ILE 1053, SER 803	ARG 1027	ASP 1028	PHE 845, ALA 844, ALA 881, ILE 804, VAL 805, LEU 802, ALA 1065, LEU 1067, PRO 1068, PHE 1047, LEU 1049, ILE 1053, TYR 1054, MET 1072, TYR 1082, VAL 1060, TYR 1059

## Conclusions

We have prepared several trimeric triaryl substituted pyridinium cations with numerous counter anions, including Br^–^, BF_4_^–^, PF_6_^–^, and CF_3_SO_3_^–^ in conventional and Muffle furnace circumstances. We have found that a solid-phase reaction has more benefits, such as a greater yield, a faster reaction time, an environmentally friendly nature, and a simple workup method. We have examined the catalytic properties of our trimeric triaryl substituted pyridinium salts (good catalytic concentration) for the one-pot synthesis of Gem-bisamide and its derivatives using conventional and muffle furnace approaches. The conventional method is fifty times faster than the solvent-free Muffle furnace condition. When compared to other reactions, a one-pot reaction with our synthesized compounds **3(a-d)** included showed excellent catalytic response. Interestingly, the candidate 4-nitrobenzyl substituted triaryl pyridinium cation with the BF_4_^–^ anion has a much higher binding interaction with the model protein active site of the VEGFR-2 kinase receptor. It was ultimately shown that these trimeric triaryl pyridinium ionic liquids would work well as anticancer medications for treating human cancer.

### Ethical approval

This article does not contain any studies with human participants or animals performed by the authors.

### Consent to participate

We comply with the ethical standards. We provide our consent to take part.

## Supplementary Information

Supplementary Information.

## Data Availability

The datasets used and/or analysed during the current study available from the corresponding author on reasonable request.
