# Supplementary material for: Catalytic response and molecular simulation studies in the development of synthetic routes in trimeric triaryl pyridinium type ionic liquids
Source: Sci Rep. 2023 Mar 17;13:4453. doi: 10.1038/s41598-023-31476-0 (PMC10023811; doi:10.1038/s41598-023-31476-0)
Supplement: Supplementary file 1 — Supplementary Information. [file 41598_2023_31476_MOESM1_ESM.docx]

**Supporting Information**

**Catalytic Response and Molecular Simulation Studies in the Development of Synthetic Routes in Trimeric Triaryl Pyridinium Type Ionic Liquids**

Ramalingam Tamilarasan^1^, Annadurai Subramani^2^, G. Sasikumar^2^, Pandurangan Ganapathi^3^, S. Karthikeyan^4^, Sasikumar Ponnusamy^5,*^, Salim Albukhaty^6^, Mustafa K. A. Mohammed^7,**^, Zaidon T. Al-aqbi^8^, Faris A. J. Al-Doghachi^9^, Duha S. Ahmed^10^, Yun Hin Taufiq-Yap^11,12,***^

^1^ Department of Chemistry, Vel Tech Multi Tech Dr. Rangarajan Dr. Sakunthala Engineering College, Avadi, Chennai, India

^2^ Department of biochemistry Dwaraka Doss Goverdhan Doss Vaishnav College Chennai- 600106, Tamilnadu, India

^3^ Department of Chemistry, Mohamed Sathak College of Arts & Science, Sholinganallur, Chennai, India

^4^ Department of Physics, Periyar University Centre for Post Graduate and Research Studies, Dharmapuri-636 701, India

^5^ Department of Physics, Saveetha School of Engineering, (SIMATS), Thandalam, Chennai 602 105, India

^6^ Department of Chemistry, College of Science, University of Misan, Maysan, Iraq

^7^ Radiological Techniques Department, Al-Mustaqbal University College, 51001 Hillah, Babylon, Iraq

^8^ College of Agriculture, University of Misan, Al-Amara, Misan 62001, Iraq

^9^ Department of Chemistry, Faculty of Science, University of Basrah, Basrah 61004, Iraq

^10^ Applied Science Department, University of Technology, Baghdad 10011, Iraq

^11^ Catalysis Science and Technology Research Centre, Faculty of Science, Universiti Putra Malaysia, Serdang 43400, Selangor, Malaysia

^12^ Faculty of Science and Natural Resources, University Malaysia Sabah, 88400 Kota Kinabalu, Sabah, Malaysia

**^*^**Corresponding Authors: [sasijanaki123@gmail.com](mailto:sasijanaki123@gmail.com) **(Dr. P. Sasikumar)**

**^**^** [mustafa_kareem97@yahoo.com](mailto:mustafa_kareem97@yahoo.com) **(Dr. M.K.A. Mohammed)**

**^***^** [taufiq@upm.edu.my](mailto:taufiq@upm.edu.my) **(Prof. Y.H. Taufiq-Yap)**

**Ligand and protein preparation:**

The 3D structures of synthesized compounds were constructed by utilizing the Maestro-11.8.012 builder panel (<https://www.schrodinger.com/releases/release-2018-4>). The ligand preparation wizard was used to add hydrogen atoms and regulate the rational bond angles, geometry, and ring conformations. The most favorable ionization state of compounds was produced with the aid of force field OPLS-2005 (optimized potentials for liquid simulations), and geometry minimization was carried out until it reached a root-mean-square deviation cutoff of 0.01 Å. The subsequent output structure of compounds was suitable for docking with the protein VEGFR-2 kinase.

The crystal structure of the VEGFR-2 kinase (PDB ID: 4AGD) was downloaded from the protein data bank (<https://www.rcsb.org/structure/4agd>). The receptor, which was fully optimized in the protein preparation wizard*,* was used to add polar hydrogen atoms, assign bond orders and protonation states, and then delete water beyond 5Å. Thereafter, by using a force field (OPLS-2005), the energy minimization of 0.03 Å RMSD was performed to reduce the steric hindrance caused by the addition of hydrogen atoms. Now the minimized VEGFR-2 receptor was suitable for docking with the supplied x, y, and z coordinates of 45.14, -7.99, and -8.72 Å, respectively, with a grid box diameter (xyz) of 30×30×30 Å. The output results were visualized with the help of the PyMol console for a virtual examination of various binding modes between compounds and receptors.

**General experimental conditions**

Preparation of 1, 3, 5-tri (pyridine-2-yl)-1, 3, 5-triazinane **1**

3.0 g of 1, 3, 5-tri (pyridine-2-yl)-1, 3, 5-triazinane (3.18 × 10^-3^ mmol; 1.0 equiv.) is mixed with 1.00 g of formaldehyde (3.347 × 10^-2^ mmol; 1.05 equiv.) and 4.6 g of K_2_CO_3_ dissolved (3.328 × 10^-2^ mmol; 1.05 equiv.) in the presence of 50 mL of CH_3_CN at room temperature stirring for 30 min. To give compound 1 in quantitative yield. Yield: 9.85 g, (97%); Semisolid; ^1^H NMR :(400 MHz, DMSO-d_6_) δ: 2.60 (s, 6H), 3.94 (s, 6H), 7.06-7.18 (d, 6H), 7.23-7.35 (t, 3H), 7.53-7.55 (t, 3H), 7.94-8.06 (d, 6H), 8.17-8.29 (t, 3H), 8.55-8.65 (d, 3H), 8.79-8.96 (d, 3H); ^13^C NMR :(100 MHz, DMSO-d_6_) δ:79.74, 121.33, 124.49, 139.20, 153.17, 161.28:MS (FAB): *m/z* 318.16; Anal. Calcd for: C_18_H_18_N_6_: C: 67.90: H: 5.70: N: 26.40; Found: C: 67.89: H: 5.85: N: 26.39.

**Solvent-free route**

Fine grinding with silica gel without solvent is used to prepare the above-mentioned reactive substrate. The solid-phase reacting substrate is blended together and separated into two parts: (i) the first half of the reacting substrate is kept in a muffle furnace at 100 °C, and (ii) the second half of the reacting substrate is kept in a domestic microwave oven. The progress of the reaction is monitored by TLC.

**General procedure for *N*-alkylation reaction**

1, 3, 5-tri (pyridine-2-yl)-1, 3, 5-triazinane 1 (6.281 ×10^-3^ mmol; 1.00 equi) is treated with benzyl bromide /4-nitrobenzyl bromide (1.916 × 10^-3^ mmol; 3.05 equiv.) in the presence of 20 mL dry acetonitrile under refluxing condition for 2-3 hours to give *N-*alkylated product of compound 2a/3a.

***N-*alkylation without the use of solvents**

Fine grinding of the above-mentioned reactive substrate with silica gel is used. The reactive substrate with solid support is separated into two parts: i) one half of the reacting substrate is held in a muffle furnace at 100 °C, while the other half is microwaved in a domestic microwave oven. TLC is used to monitor the reaction's progress.

1, 3, 5-tri (benzyl pyridinium bromide-2-yl) -1, 3, 5-triazinane**(2a)**

Yield: 4.7 g (90%); MP: 242^0^C;^1^H NMR :(400 MHz, DMSO-d_6_) δ: 1.7 (s, 6H), 6.96-7.12 (m, 9H), 7.39-7.51 (m, 15H), 7.76 (d, 3H); ^13^C NMR :(100 MHz, DMSO-d_6_) δ: 56.2, 80.7, 120.3, 125.1, 128.4, 138.4, 152.2, 153.8, 163.8, 164.3, 168.8: MS (FAB): *m/z* 831.48; Anal. Calcd for: C_39_H_39_Br_3_N_6_: C: 56.34: H: 4.73: N: 10.11; Found: C: 56.28: H: 4.69: N: 10.10.

1, 3, 5-tri (nitrobenzyl pyridinium bromide-2-yl) -1, 3, 5-triazinane **(3a)**

Yield: 5.85g, (96%); MP: 260^0^C; ^1^H NMR: (400 MHz, DMSO-d_6_)δ: 1.73 (s, 6H), 2.6 (s, 6H), 7.21-7.24 (m, 9H), 7.36-7.40 (m, 6H), 7.73-7.77 (d, 9H), 7.79 (d, 6H),; ^13^C NMR :(100 MHz, DMSO-d_6_) δ: 56.04, 80.95, 125.48, 129.58, 138.53, 145.29, 152.29, 153.89, 163.83, 164.40, 168.96:MS (FAB) : *m/z* 966.47; Anal. Calcd for: C_39_H_36_Br_3_N_9_O_6_: C: 48.47: H: 3.75: N: 13.04; Found: C: 48.42: H: 3.72: N: 13.03.

General procedure for anion exchange reaction was carried out from benzyl/nitrobenzyl bridged trimeric pyridinium bromide 2a/3a (2.65 × 10^-4^ mmol; 1.0 equiv.) is treated with various counter anion containing inorganic salt such as NaBF_4_, K_4_PF_6_, and LiCF_3_SO_3_ (6.208 × 10^-2^ mmol; 2.05 equiv.) is dissolved in 20 mL of deionized water at room temperature stirring for 2 hours to give anion exchange product of compound 2a/3a(b-d) in 90-97% yield. Metallic bromide and benzyl/nitrobenzyl-bridged trimeric pyridinium salts are soluble in water, so separation is not easier. Under these circumstance, Soxhlet extraction is used in for purification dry THF for 1h refluxtion. Anion exchanged reaction is confirmed by checked by aqueous AgNO_3_ solution.

1, 3, 5-tri (benzyl pyridinium hexafluorophosphate-2-yl) -1, 3, 5-triazinane **(2b)** Yield: 0.28g, (91%); MP: 225 ^0^C;^1^H NMR :(400 MHz, DMSO-d_6_) δ: 1.6 (s, 6H), 6.94-7.10 (m, 9H), 7.37-7.49 (m, 15H), 7.74 (d, 3H); ^13^C NMR :(100 MHz, DMSO-d_6_) δ: 56.0, 80.5, 120.1, 124.9, 128.2, 138.2, 152.0, 153.6, 163.6, 164.1, 168.6: MS (FAB): *m/z*1198.92; Anal. Calcd for: C_59_H_44_F_12_N_2_O_8_P_2_: C: 59.11: H: 3.70: N: 2.34; Found: C: 59.05: H: 3.66: N: 2.28.

1, 3, 5-tri (benzyl pyridinium tetrafluoroborate-2-yl) -1, 3, 5-triazinane**(2c)**

Yield: 0.26g, (94%), MP: 230^0^C;^1^H NMR :(400 MHz, DMSO-d_6_) δ: 1.5 (s, 6H), 6.93-7.09 (m, 9H), 7.36-7.48 (m, 15H), 7.73 (d, 3H); ^13^C NMR :(100 MHz, DMSO-d_6_) δ: 55.0, 80.4, 120.0, 124.8, 128.1, 138.1, 151.09, 153.5, 163.5, 164.0, 168.5: MS (FAB): *m/z* 1082.6; Anal. Calcd for: C_59_H_44_B_2_F_8_N_2_O_8_: C: 65.46: H: 4.10: N: 2.59; Found: C: 65.39: H: 4.06: N: 2.50

1, 3, 5-tri (benzyl pyridinium trifluoromethanesulfonate-2-yl) -1, 3, 5-triazinane**(2d)**

Yield: 0.3g, (96%): MP: 178 ^0^C;^1^H NMR :(400 MHz, DMSO-d_6_) δ: 1.49 (s, 6H), 6.92-7.08 (m, 9H), 7.35-7.47 (m, 15H), 7.72 (d, 3H); ^13^C NMR :(100 MHz, DMSO-d_6_) δ: 54.09, 80.3, 119.09, 124.7, 128.0, 138.0, 151.08, 153.4, 163.4, 163.09, 168.4: MS (FAB): *m/z* 1207.17; Anal. Calcd for: C_61_H_44_F_6_N_2_O_14_S_2_: C: 60.69: H: 3.67: N: 2.32; Found: C: 60.63: H: 3.58: N: 2.26.

1, 3, 5-tri (nitrobenzyl pyridinium hexafluorophosphate-2-yl) -1, 3, 5-triazinane**(3b)**

Yield: 0.3g, (93%), MP: 230^0^C;^1^H NMR: (400 MHz, DMSO-d_6_)δ: 1.72 (s, 6H), 2.5 (s, 6H), 7.20-7.23 (m, 9H), 7.35-7.39 (m, 6H), 7.72-7.76 (d, 9H), 7.78 (d, 6H),; ^13^C NMR :(100 MHz, DMSO-d_6_) δ: 56.03, 80.94, 125.47, 129.57, 138.52, 145.28, 152.28, 153.88, 163.82, 164.39, 168.95:MS (FAB) : *m/z* 1232.93; Anal. Calcd for: C_62_H_42_F_12_N_2_O_8_P_2_: C: 60.40: H: 3.43: N: 2.27; Found: C: 60.34: H: 3.40: N: 2.25.

1, 3, 5-tri (nitrobenzyl pyridinium tetrafluoroborate-2-yl) -1, 3, 5-triazinane **(3c)**

Yield: 0.29g, (96%), MP: 253^0^C;^1^H NMR: (400 MHz, DMSO-d_6_)δ: 1.71 (s, 6H), 2.4 (s, 6H), 7.19-7.22 (m, 9H), 7.34-7.38 (m, 6H), 7.71-7.75 (d, 9H), 7.77 (d, 6H),; ^13^C NMR :(100 MHz, DMSO-d_6_) δ: 56.02, 80.93, 125.46, 129.56, 138.51, 145.27, 152.27, 153.87, 163.81, 164.38, 168.94: MS (FAB) : *m/z* 1116.61; Anal. Calcd for: C_62_H_42_B_2_F_8_N_2_O_8_: C: 66.69: H: 3.79: N: 2.51; Found: C: 66.63: H: 3.76: N: 2.50.

1, 3, 5-tri (nitrobenzyl pyridinium trifluoromethanesulfonate-2-yl) -1, 3, 5-triazinane**(3d)**

Yield: 0.31g, (97%), MP: 219^0^C;^1^H NMR: (400 MHz, DMSO-d_6_)δ: 1.70 (s, 6H), 2.3 (s, 6H), 7.18-7.21 (m, 9H), 7.33-7.37 (m, 6H), 7.70-7.74 (d, 9H), 7.76 (d, 6H),; ^13^C NMR :(100 MHz, DMSO-d_6_) δ: 56.01, 80.92, 125.45, 129.55, 138.50, 145.26, 152.26, 153.86, 163.80, 164.37, 168.93: MS (FAB) : *m/z* 1243.16; Anal. Calcd for: C_64_H_44_F_6_N_2_O_14_S_2_: C: 61.93: H: 3.41: N: 2.26; Found: C: 61.84: H: 3.37: N: 2.20.

**General procedure for the preparation of substituted Gem-bisamide derivatives:**

Simple / substituted aryl aldehyde (9.423 × 10^-3^ mmol; 1.0 equiv.) is mixed with benzamide (1.931 × 10^-2^ mmol; 2.05 equiv.) and optimized catalyst concentration of benzyl / nitrobenzyl bridged trimeric pyridinium bromide 3a (6.208 × 10^-2^ mmol) is added in the presence of 40 mL of dry CH_3_CN for 05-35 minutes under refluxing condition to give substituted Gem-bisamide derivatives 4(a-e) in 89-99% after purification. All the synthesized Gem-bisamide derivatives are throughly characterized by spectral and analytical data and verified with literature values.

**Preparation of substituted Gem-bisamide derivatives without the use of solvents**

The above-mentioned reactive substrate is thoroughly mixed with silica gel. The solid support reacting substrates is separated into two sections: one half is held in a muffle furnace at 100°C, while the other half is microwave in a domestic microwave oven. TLC is used to monitor the progress of both the reactions.

*N,N*-Benzylidenebisbenzamide**4a**

Yield : 0.5g, (91%), 236-238^0^C; ^1^H NMR:(400 MHz, DMSO-d_6_) δ: 7.03-7.07 (t, 1H), 7.31-7.35 (t, 1H), 7.38–7.42 (t, 2H), 7.48-7.51 (t, 6H), 7.55-7.59 (t, 2H), 7.91-7.93 (d, 4H), 9.04-9.06 (d, 2H);^13^C NMR:(100 MHz, DMSO-d_6_) δ: 59.1, 126.9, 127.9, 128.2, 128.8, 132.1, 134.1, 140.73, 166.0;MS (FAB) : *m/z* 330.14; Anal. Calcd for: C_21_H_18_N_2_O_2_: C: 76.34: H: 5.49: N: 8.48; Found: C: 76.33: H: 5.45: N: 8.48.

*N,N’*-(4-Methoxybenzylidene) bisbenzamide**4b**

Yield : 0.5g, (87%), 232-234^0^C; ^1^H NMR:(400 MHz, DMSO-d_6_) δ: 3.71 (s, 3H), 6.97-7.01 (t, 1H), 7.35-7.59 (m, 10H), 7.82-7.84 (d, 4H), 8.59-8.61 (d, 2H);^13^C NMR :(100 MHz, DMSO-d_6_) δ: 55.3, 58.9, 113.7, 127.4, 128.4, 131.6, 132.6, 134.1, 159.1, 166.2; MS (FAB) : *m/z* 360.41; Anal. Calcd for: C_22_H_20_N_2_O_3_: C: 73.32: H: 5.59: N: 7.77; Found: C: 73.24: H: 5.54: N: 7.76.

*N, N’*-(4-Hydroxybenzylidene) bisbenzamide**4c**

Yield : 0.5 g, (90 %), 228-230 ^0^C; ^1^H NMR :(400 MHz, DMSO-d_6_) δ: 6.18 (d, 1H), 7.24-7.50 (m, 8H), 7.78 (d, 1H), 7.92-8.02 (m, 2H), 8.88 (d, 1H); ^13^C NMR :(100 MHz, DMSO-d_6_) δ: 53.8, 113.9, 116.8, 122.9, 125.1, 126.9, 127.4, 128.6, 128.8, 128.9, 130.2, 130.4, 142.7, 147.3 and 149.4; MS (FAB) : *m/z* 346.38; Anal. Calcd for: C_21_H_18_N_2_O_3_: C: 72.82: H: 5.24: N: 8.09; Found: C: 72.75: H: 5.19: N: 8.08.

*N, N’*-(4-Nitrobenzylidene) bisbenzamide**4d**

Yield : 0.5g, (85%), 266-268 ^0^C; ^1^H NMR :(400 MHz, DMSO-d_6_) δ: 7.07-7.11 (t, 1H), 7.49-7.60 (m, 6H), 7.76-7.78 (d, 2H), 7.94-7.96 (d, 4H), 8.27-8.29 (d, 2H), 9.24-9.26 (d, 2H);^13^C NMR :(100 MHz, DMSO-d_6_) δ: 56.6, 123.9, 124.0, 128.0, 128.5, 128.8, 133.4, 147.0, 147.5, 166.3;MS (FAB) : *m/z* 375.38; Anal. Calcd for: C_21_H_17_N_3_O_4_: C: 67.19: H: 4.56: N: 11.19; Found: C: 67.13: H: 4.52: N: 11.18.

*N, N’*-(3-Nitrobenzylidene) bisbenzamide**4e**

Yield : 0.5 g, (89 %), 193-195^0^C; ^1^H NMR :(400 MHz, DMSO-d_6_) δ: 7.07-7.10 (t, 1H), 7.49-7.60 (m, 6H), 7.69-7.73 (t, 1H), 7.93-7.96 (m, 5H), 8.21-8.23 ( d, 1H), 8.34 (s, 1H), 9.26-9.28 (d, 2H);^13^C NMR :(100 MHz, DMSO-d_6_) δ: 59.3, 121.8, 123.2, 128.0, 128.8, 130.4, 132.2, 134.0, 142.8, 148.2, 166.3;MS (FAB) : *m/z* 375.38; Elemental analysis : C_21_H_17_N_3_O_4_ Cald. C: 67.19; H: 4.56; N: 11.19; Found: C: 67.13: H: 4.52: N: 11.18.


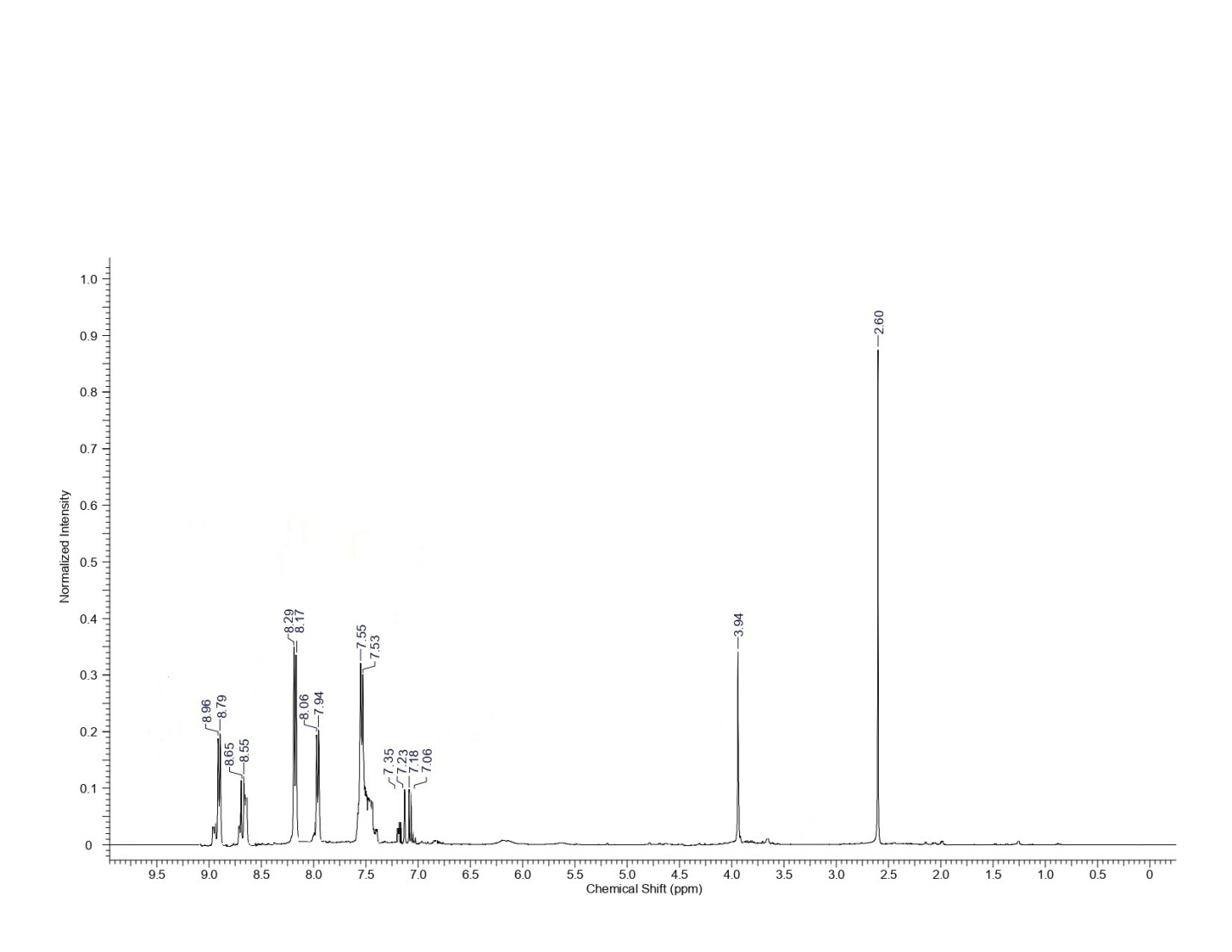


**Fig-1:**^1^H NMR Spectrum of 1, 3, 5-tri (pyridine-2-yl)-1, 3, 5-triazinane**1**


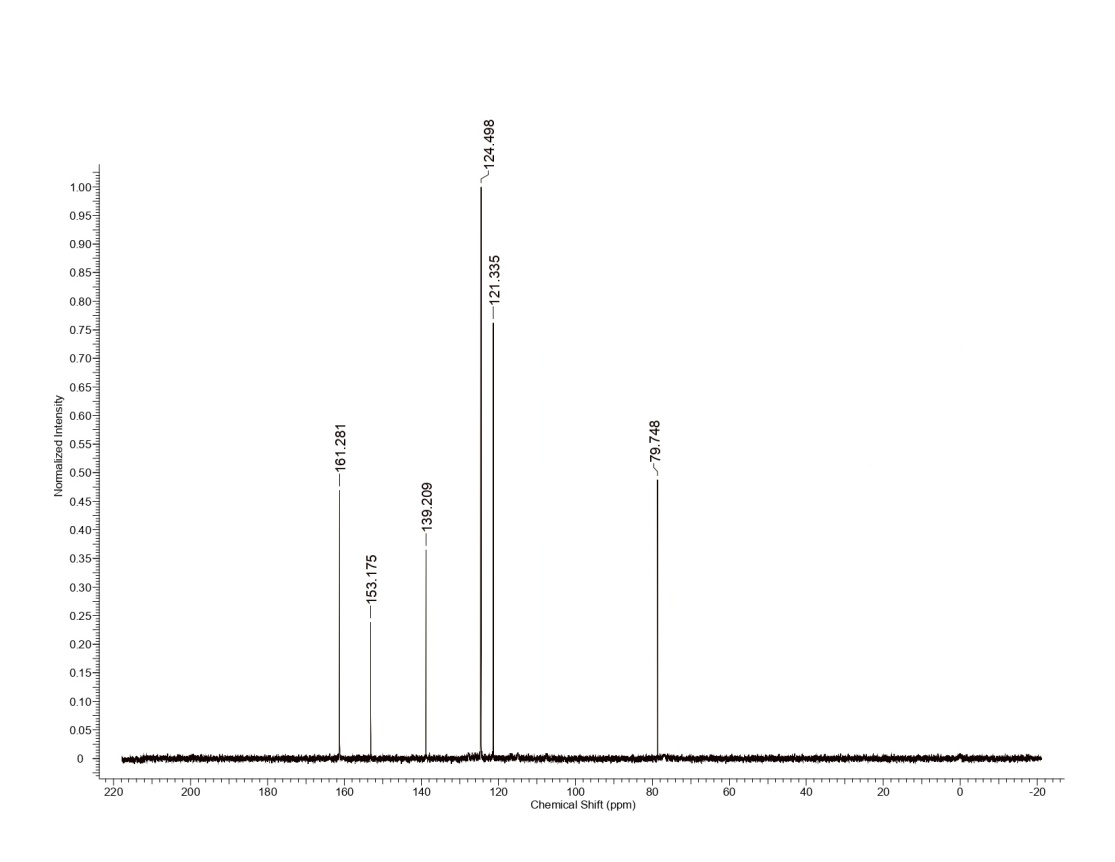


**Fig-2:**^13^C NMR Spectrum of 1, 3, 5-tri (pyridine-2-yl)-1, 3, 5-triazinane **1**


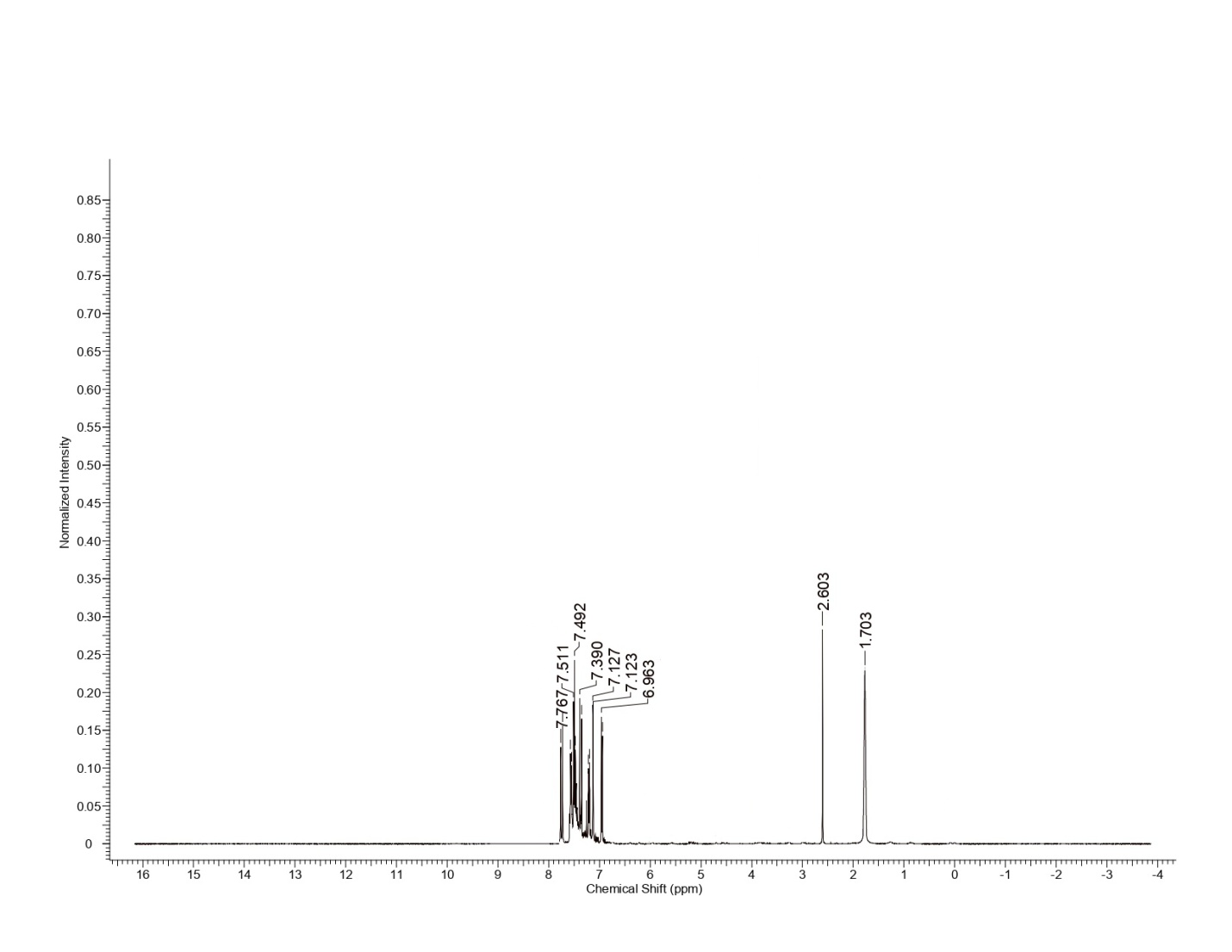


**Fig-3:**^1^H NMR Spectrum of 1, 3, 5-tri (benzyl pyridinium bromide-2-yl) -1, 3, 5-triazinane **2a**


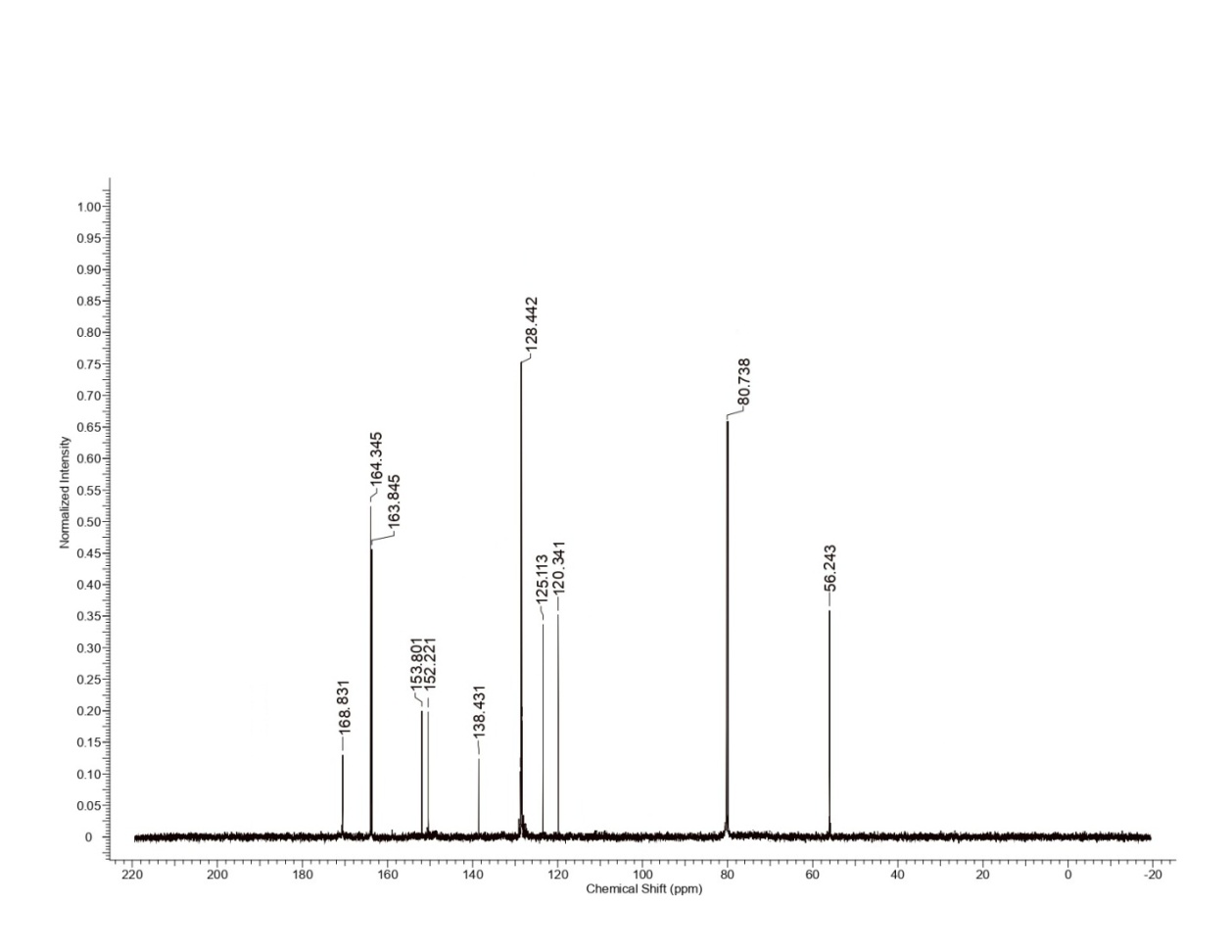


**Fig-4:**^13^C NMR Spectrum of 1, 3, 5-tri (benzyl pyridinium bromide-2-yl) -1, 3, 5-triazinane **2a**


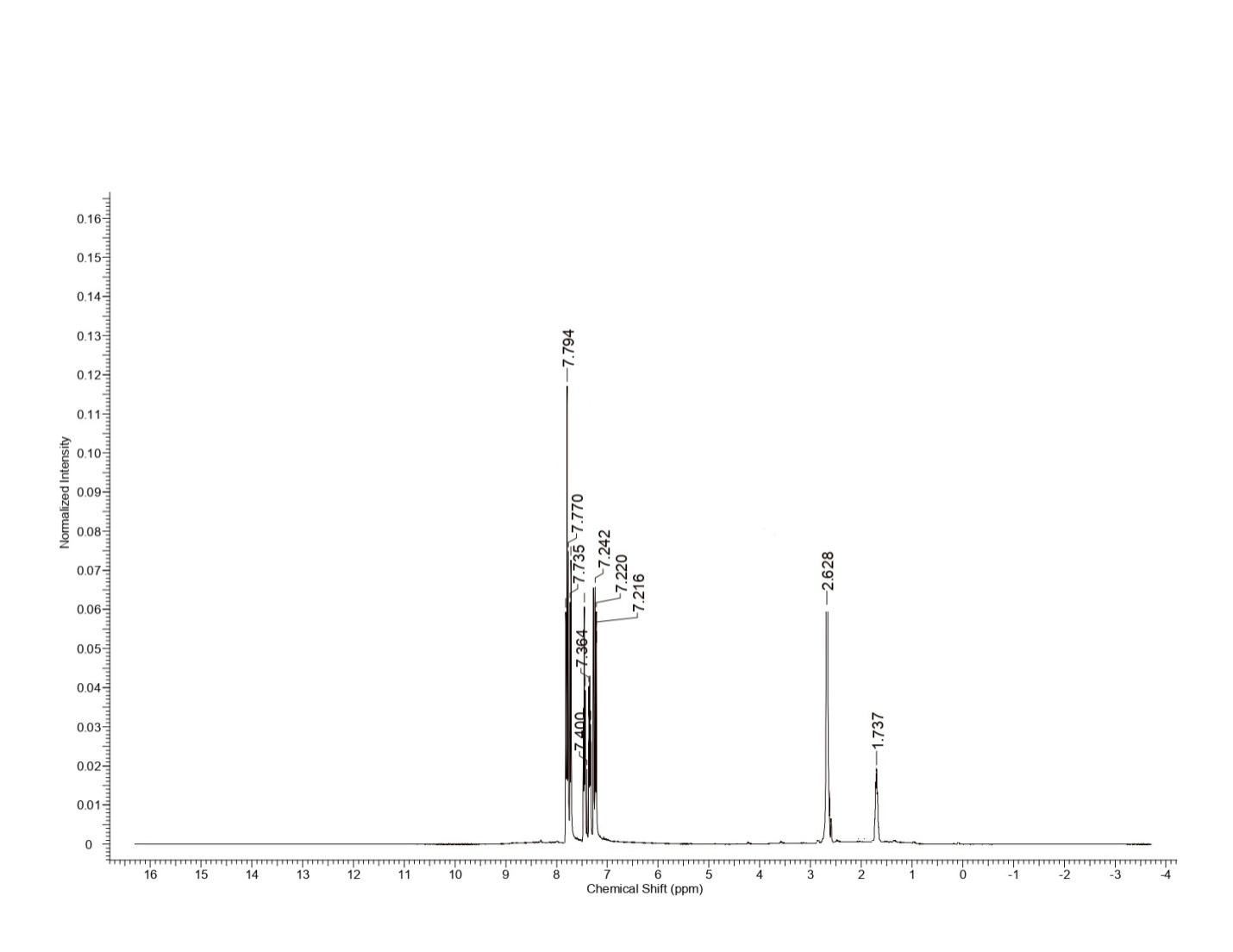


**Fig-5:**^1^H NMR Spectrum of 1, 3, 5-tri (nitrobenzyl pyridinium bromide-2-yl) -1, 3, 5-triazinane**(3a)**


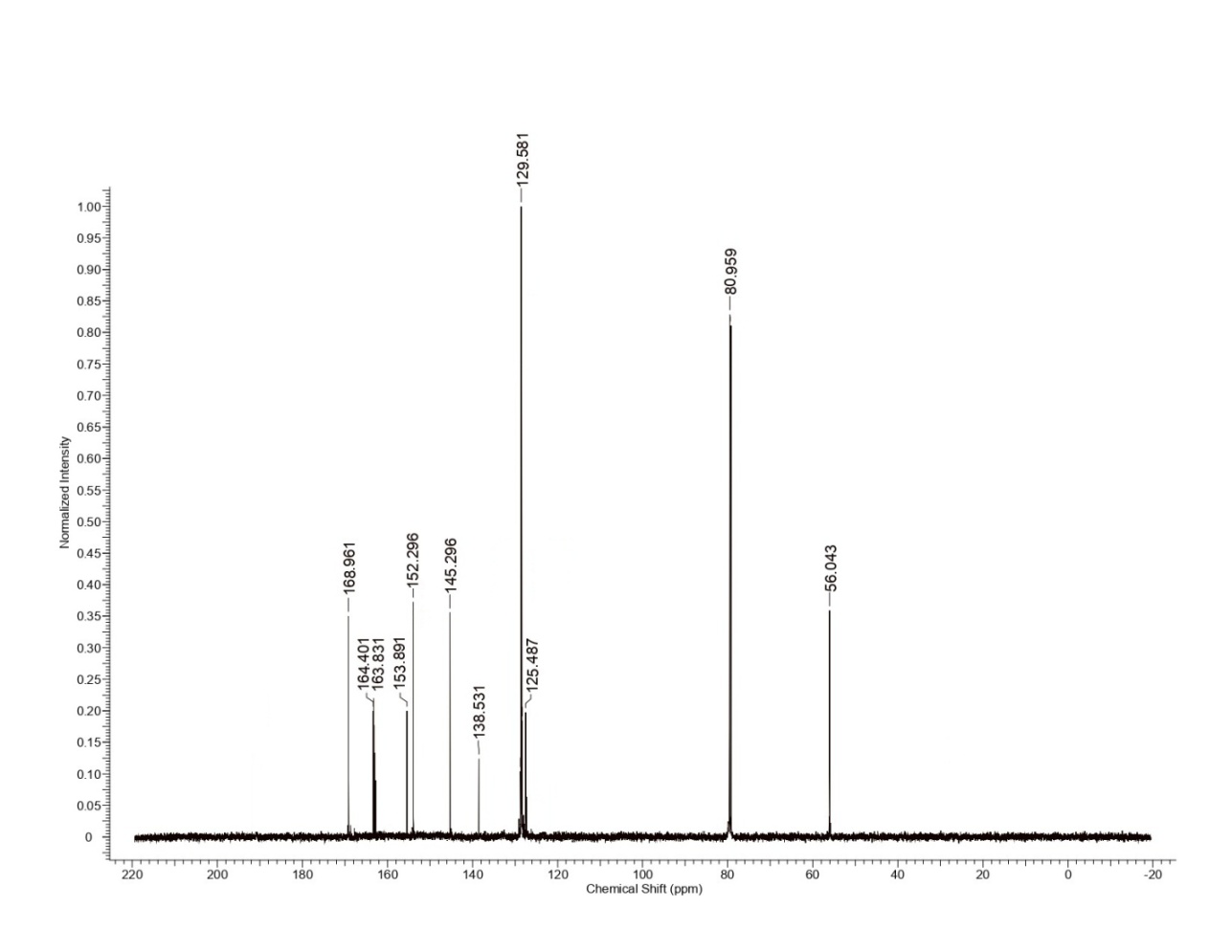


**Fig-6:**^13^C NMR Spectrum of 1, 3, 5-tri (nitrobenzyl pyridinium bromide-2-yl) -1, 3, 5-triazinane**(3a)**
